# AI-deploying organizations are key to addressing ‘perfect storm’ of AI risks

**DOI:** 10.1007/s43681-022-00163-7

**Published:** 2022-05-24

**Authors:** Caitlin Curtis, Nicole Gillespie, Steven Lockey

**Affiliations:** 1 School of Business, The University of Queensland, Brisbane, QLD 4072 Australia; 2 Centre for Policy Futures, The University of Queensland, Brisbane, QLD 4072 Australia; 3 Centre for Corporate Reputation, University of Oxford, Oxford, UK

**Keywords:** Artificial intelligence, Ethics, Trustworthy AI, Risks, Organization

## Abstract

We argue that a perfect storm of five conditions heightens the risk of harm to society from artificial intelligence: (1) the powerful, invisible nature of AI, (2) low public awareness and AI literacy, (3) rapid scaled deployment of AI, (4) insufficient regulation, and (5) the gap between trustworthy AI principles and practices. To prevent harm, fit-for-purpose regulation and public AI literacy programs have been recommended, but education and government regulation will not be sufficient: AI-deploying organizations need to play a central role in creating and deploying trustworthy AI in line with the principles of trustworthy AI, and taking accountability to mitigate the risks.

## Introduction

Given the increasingly ubiquitous nature of artificial intelligence (AI) systems and their growing incorporation into everything from social media to virtual assistants, most members of the public are now likely to interact with AI in some form daily, whether knowingly or not [1]. There is undeniable potential for AI and related technologies to address global challenges and beneficially contribute to advancing society [2], for example AI has the potential to improve diagnostic predictions and decision-making in areas such as healthcare [3], weather [4], and agriculture [5]. However, the risks from AI systems are equally undeniable. We argue there is a ‘perfect storm’ of conditions related to AI systems that significantly heightens the risk of harm to society, and organizations are key to proactively averting the storm. The ‘perfect storm’ metaphor depicts a rare combination of events that creates an unusually bad situation. It has been used to describe previous global conditions, such as the global financial crisis, whereby an ‘underestimation of risk, opacity, interconnection, and leverage, all combined to create the perfect (financial) storm’ [6].

**Fig. 1:**
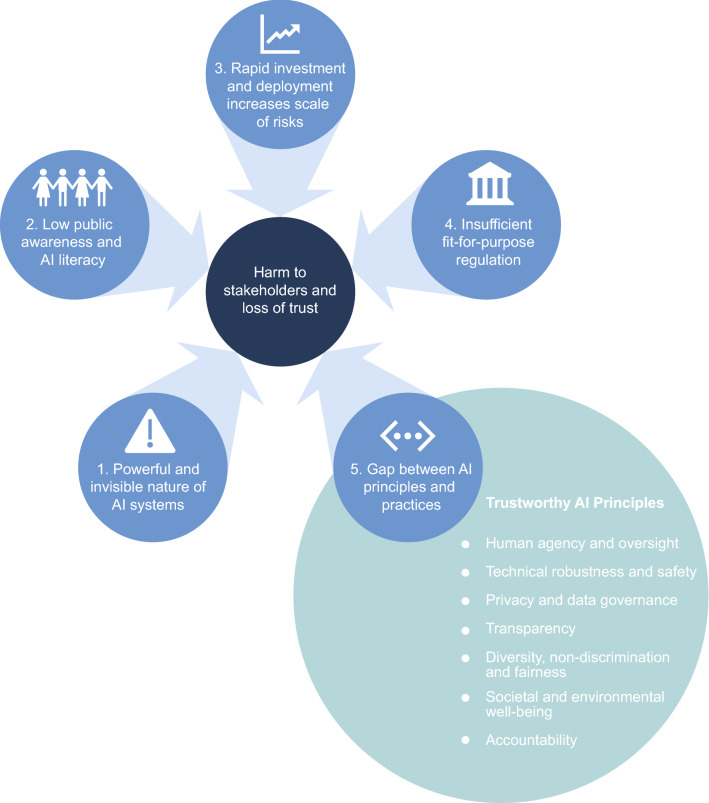
A confluence of conditions heightening risk of harm to society from AI systems. (Trustworthy principles drawn from European Commission 2019)

Specifically, we argue that a perfect storm augmenting the risk of societal harm from AI systems is emerging due to the confluence of five conditions: (1) the powerful, invisible nature of AI systems, (2) low public awareness and AI literacy, (3) the rapid scale of AI system deployment, (4) insufficient regulation, and (5) the gap between AI principles and practices for trustworthy AI systems. Figure 1 illustrates a model of this perfect storm. We explain each of the five conditions contributing to this perfect storm in turn, and how they work synergistically to augment risks. Although we present these conditions as a numbered list, there is no hierarchy to the ordering: each condition is important and plays a role in augmenting risk. We conclude with a discussion of the practical and policy implications of this perfect storm, and the central role that organizations involved in the development and/or use of AI systems must play if this storm is to be averted.

In so doing, we reference evidence on public perceptions of AI risks and governance challenges drawn from our recent multi-country survey on public trust and attitudes towards AI systems [7]. This survey collected data from over 6,000 people from five Western countries—USA (*N* = 1223), Canada (*N* = 1229), UK (*N* = 1200), Germany (*N* = 1202) and Australia (*N* = 1200) using nationally representative research panels. The samples were matched against census data for each country with respect to age, gender, and geographic location. The survey questions were based on established measures either directly adopted or adapted from prior peer reviewed articles or prior published public attitude surveys. Survey questions were professionally translated and back-translated for the French (Canadian) and German samples. Further details of the survey can be found in the full report [7]. We also draw on other recent surveys and empirical evidence where relevant.

## Conditions heightening the risk of harm from AI systems

1. ***The powerful and invisible nature of AI systems***

Although all emerging technologies carry some form of risk, AI systems present unique and well-identified challenges (Fig. 1). In part, these unique risks stem from the ‘black box’ nature of AI systems, which makes them problematic to explain and understand [8, 9], and in turn hampers accountability-at-large [10]. In instances where the inner workings of AI systems that are used for decision-making or other important processes are opaque and not easily viewed or understood by users or other parties, errors may not be perceived by users or the organizations developing or deploying the AI systems. Biased outcomes of opaque AI systems [11] have resulted in discrimination [12] and harm, highlighting the risk that AI systems can unintentionally codify, compound, and promulgate existing societal biases evident within datasets [13], through ‘data cascades’ [14] and runaway feedback loops [15]. This underscores the need to develop ways to detect errors early. Furthermore, those most impacted by AI systems may belong to the most vulnerable groups with the least power and agency [16], and the least awareness of how AI is being used to make decisions about them. This highlights the need for meaningful consultation with voices that are often underrepresented.

The powerful nature of AI systems, left unchecked, can threaten societal values and constitutional rights, including autonomy, privacy [17], and democracy, giving rise to power imbalances when deployed at scale [18, 19]. For example, facial recognition systems can be used to target surveillance of ethnic minorities [20, 21]. A 2019 report estimated that at least 75 countries were actively using AI technologies for surveillance purposes, including 51% of advanced democracies [22]. During COVID-19, tensions between beneficial data sharing and concerns around surveillance and privacy have emerged with contact tracing apps mandated by many governments [23]. Strong temptations for governments and organizations to gather and access as much data as they can [18, 24] for policy or profit, raises concerns about the possibility of mission creep [25].

These risks are augmented by the invisible and ubiquitous nature of AI systems, obscuring when and where these systems are in use. For example, it can be difficult to determine whether a decision is being made by technology or a person, and therefore use of AI systems may go unnoticed, making it harder to regulate and for citizens to play an active role in mitigating risks. AI systems may play a seemingly ‘invisible’ role in a variety of contexts where they are not easily noticed or recognized by most people, such as email spam filters, digital curation systems that recommend products during online shopping, facial recognition systems, and the assorted algorithms that determine our insurance or credit risk—often behind the scenes. The influence of algorithmic decision-making is likely to be underestimated [26], however there is mounting evidence that AI systems can influence important life decisions, such as dating, job selection and political judgements [27, 28].

**Fig. 2:**
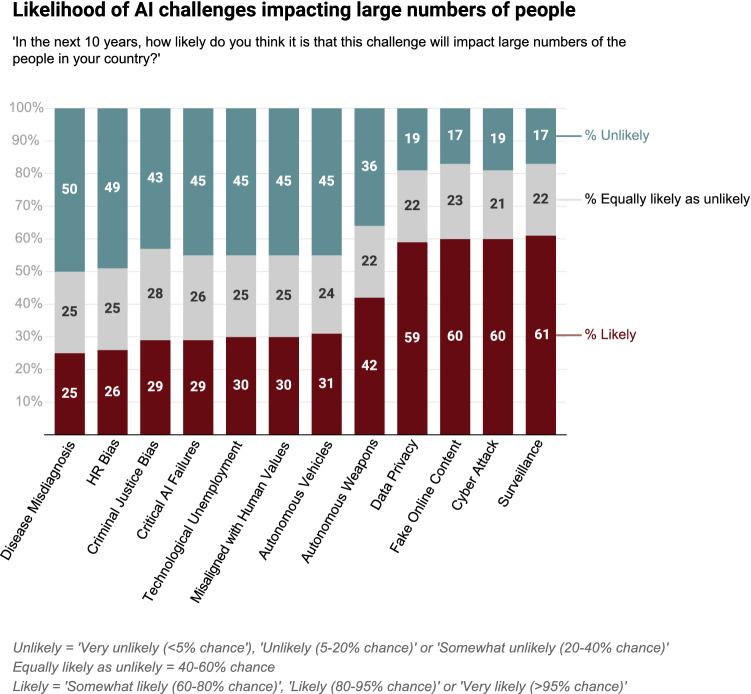
Perceptions of the impact of AI challenges on society (From Gillespie, Lockey, and Curtis, [7])

Our survey data provides clear evidence of wide-ranging concerns around the risks related to AI systems [7]. The majority of respondents (59–61%) believe four key challenges relating to AI are likely to impact large numbers of citizens in the next decade: mass surveillance, AI-enabled fake online content, cyber-attacks, and data privacy breaches (Fig. 2). A further 43–50% of respondents believe that AI-enabled disease misdiagnosis, bias, technological unemployment, and critical AI system failures will impact their communities. Similar threats relating to security, verification, “deep fake” videos, mass surveillance, and advanced weaponry were also identified by stakeholders in a recent World Economic Forum Report [29].

2. ***Low public awareness and AI literacy***

Low public understanding and awareness about AI is contributing to a deepening ‘digital divide’, hindering full and meaningful evaluation of how and to what degree AI systems are impacting individuals and communities (Fig. 1). This constrains the public’s capacity to meaningfully engage with policy and governance proposals and contribute to the mitigation of risks. The gap in the integration of the trustworthy AI principle of transparency contributes to this low literacy and awareness of AI throughout the general population and has led to calls for the promotion of greater transparency in all aspects of AI design, extending to the intentions of the system creators and disclosures of funding sources [30].

**Fig. 3:**
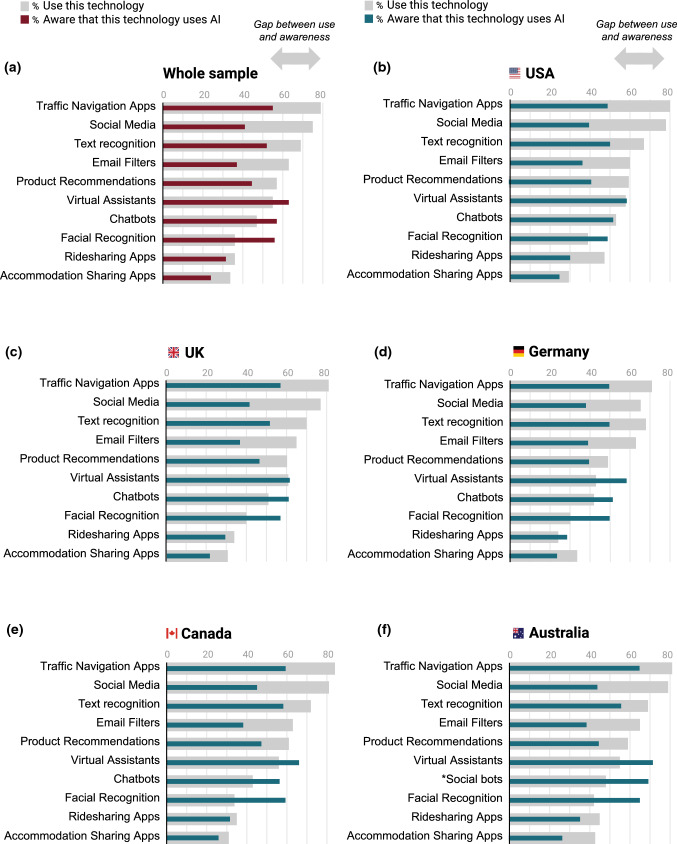
Survey results of the gap between the proportion of people using an AI-enabled technology and the proportion of people who are aware that the technologies use AI. The areas (in grey) from the end of the colored lines to the end of the grey bars indicate a gap between the use of a technology and awareness that it uses AI. (Adapted from Gillespie, Lockey, and Curtis, [7])

Our survey revealed that public awareness of AI is surprisingly low, with only 62% having seen, read, or heard anything about AI and the majority self-reporting low understanding of AI. Furthermore, when presented with a range of common AI applications, many people were not aware that the technology used AI (Fig. 3). As shown in Fig. 3, use of a technology was not always sufficient to provide a meaningful understanding of whether the technology utilizes AI. Disparity between levels of technology use and AI awareness was especially pronounced for social media and email filters: for example, 75% of people across countries reported using social media but only 41% were aware that the technology used AI (Fig. 3a). This low awareness about when, where, and how data are being gathered by and used in AI systems—even in the context of familiar everyday applications such as email filters and ride-sharing apps—is broadly consistent with prior surveys [31, 32]. We note trends in AI awareness were broadly similar among the five countries, and people generally reported less awareness of AI use in embedded technologies (e.g. social media, email filters) than in embodied technologies (e.g. where voice is used, such as virtual assistants and chatbots) (Fig. 3a–f).

When combined with low levels of understanding, the ‘behind-the-scenes’ nature of AI systems results in their use often going unchecked and unchallenged. People may never know if an algorithm made an unfair, biased, or faulty decision or recommendation about them. This augments the risks around privacy and informed consent, particularly as the data that underpin AI systems expands from standard records and organization-client interactions to social data, location data, and information collected from sources such as wearable devices, smart home systems, and digital assistants. As algorithms increasingly use these data to customize and define our choices, the options can become increasingly personalized with the goal of influencing our decisions. It has also been noted that our habitual use of these devices can lead to unquestioning acceptance [33]. This raises questions about the extent to which people are aware that they are being nudged—and how and why they are being nudged by algorithms [34].

3. ***The rapid investment and deployment of AI systems increases the scale of risks***

The growing data economy comprises the production, distribution and consumption of digital data [35]. The data economy is fueled by an increase in the volume, variety, and speed at which data are being produced and consumed [36]. Investment in AI and the data economy has increased exponentially, and all sectors of the global economy are now rapidly deploying AI. The global investment in AI has accelerated through the COVID-19 pandemic, increasing 40% from 2019 to 2020 [37]. At the same time there has been a rapid increase in the deployment of AI systems, particularly amongst large firms, with more companies deploying or piloting their own AI projects (57% in 2020, up from 44% in 2018) [38].

AI systems are also becoming more widely adopted in the public sector. Many governments rely on commercial companies for AI-enabled tools and technology, such as products for image recognition, language processing, and other applications, which may be scaled up for use by multiple state actors, increasing their reach and influence. Use of AI systems in the public sector has unique challenges including the potential for unintended consequences on millions of citizens, the potential to disproportionately impact vulnerable communities, and the integration into essential services where there may be little or no opportunity to opt out [39, 40]. Taken together, this rapid deployment in private and public domains increases the magnitude and impact of risks to citizens and society (Fig. 1).

4. ***Insufficient fit-for-purpose regulation to govern risks***

**Fig. 4:**
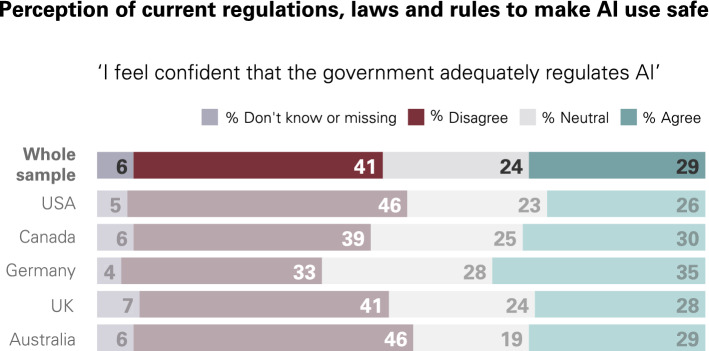
Survey results on public confidence in government regulation of AI (From Gillespie, Lockey, and Curtis, [7])

Despite the exponential uptake and use of AI, the regulation of AI systems is lagging behind. Citizens do not believe current regulation is fit-for-purpose to govern the risks associated with AI. 63% of our survey respondents disagreed or were unsure whether current safeguards are sufficient to make AI use safe, with 41% unconvinced that the government adequately regulates AI (Fig. 4). Citizens are not alone in their concerns. In a recent survey of over 1,578 technology employees, the large majority agreed the government should regulate AI and that the tech industry is too powerful [41].

Although some aspects of AI and data use are covered by existing regulatory and human rights frameworks in certain jurisdictions (such as non-discrimination laws and data protection acts), regulation to govern the risks of AI systems has been criticized for lagging behind the technology [42]. The global nature of AI system use and data sharing means that it often transcends national borders, limiting the applicability of jurisdiction-specific regulation. Since the launch of the Pan-Canadian Strategy in 2017, governments and organizations are bringing forward proposals and frameworks for AI governance and declarations of their commitment to responsible and ethical approaches to AI. However, to date, these frameworks mostly focus on providing guidance on ethical and trustworthy AI principles.

5. ***The gap between AI principles and practices for trustworthy AI systems***

The gap in the integration of the trustworthy AI principles (such as data privacy and security) are contributing to the risks relating to the rapid deployment of AI systems. One example of this can be seen in the public—private partnerships that are supporting rapid progress in the field of healthcare AI, including fields such as radiology, robotic surgery, and diagnostic imagery [43]. As a result of these partnerships, data are often controlled by private entities and/or public–private partnerships and has sometimes resulted in poor protection of privacy. In one example, the UK’s Royal Free London NHS Foundation Trust established patient data sharing with DeepMind to develop machine learning based management tools [44], however the stored patient data were later moved to the United States when DeepMind was acquired by Google [45]. Furthermore, mechanisms to deidentify or anonymize sensitive patient data may be challenged by new algorithms that have successfully reidentified these types of data –increasing the risk to patient data security in these arrangements [43]. Opportunities to repurpose existing sensitive patient datasets for monetary gain or other types of advantage can also create conflicting goals and motivations for data custodians and threaten data privacy. Likewise, in the financial sector, large technology companies are increasingly leveraging their access to extensive amounts of customer data into AI driven models to provide financial services [46]. This creates concerns about data privacy, and how the collection, storage and use of personal data may be exploited for commercial gain [47].

In response to the significant concerns and risks associated with AI systems, a proliferation of AI ethical frameworks has been produced. The European Commission [48] identified trustworthy AI as being robust, lawful, and ethical, and outlined seven central principles for trustworthy AI systems: (1) human agency and oversight, (2) technical robustness and safety, (3) privacy and data governance, (4) transparency, (5) fairness, non-discrimination, and diversity, (6) societal and environmental wellbeing, and (7) accountability (Fig. 1). Many subsequent AI ethics frameworks draw on these ideals, with an emerging convergence on a set of principles [49].

Our survey reveals strong public endorsement for the principles and practices of ethical and trustworthy AI, and desire that they will be upheld. Approximately 95% of survey respondents across the five countries view each of the seven principles above, and their associated practices, as important for trust. However, a gap has been identified between these seven trustworthy AI principles and what is happening in the everyday development and deployment of AI in practice [48]. Many organizations lack maturity and understanding in implementing ethical and trustworthy AI principles [50]. This gap is highly problematic as the existence of AI ethics frameworks signals that the challenges and risks of AI are being managed, when in reality this is often not the case.

## Implications and recommendations: policy, AI literacy, and increased organizational responsibility

To date, solutions to mitigating and averting this ‘perfect storm’ of AI system risks have focused largely on strengthening regulation and increasing public AI literacy and education [51–53]. Our survey insights concur with the importance of these two approaches. In relation to regulation, we find 81% of respondents expect some form of external AI regulation, with the majority supporting independent AI regulation by government or existing regulators.

In relation to public awareness and understanding, we find 82% of people across the five countries want to learn more about AI. Artificial intelligence literacy can be defined as *“a set of competencies that enables individuals to critically evaluate AI technologies; communicate and collaborate effectively with AI; and use AI as a tool online, at home, and in the workplace”* [30]. Increasing public AI literacy, understanding, and awareness of AI supports meaningful consultation with voices that are often overlooked. Furthermore, it supports increased participatory approaches and diversity by providing the foundations for increased involvement from domain experts and other disciplines such as the humanities and social science. As a starting point, our survey identifies several AI technologies with a significant gap between use and awareness, especially social media, where there is very low public awareness around its underlying utilization of AI, despite high numbers of people engaging with it. These technologies would benefit from immediate targeted AI awareness resources to support increased public AI literacy.

A critical element that is rarely emphasized is the need for organizations and industry to urgently step up and play a proactive role in ensuring the AI systems they develop and deploy are trustworthy and ethical, and providing assurances of this to stakeholders [50]. Trustworthy behavior in organizations and industry is required because the law will rarely be able to keep abreast of rapid technology advances. Even when regulations are established, edge cases—rare problems or situations that typically occur only at an extreme (maximum or minimum) operating parameter—can still be challenging from a regulatory perspective. With few exceptions, AI systems are developed within and by organizations, whether tech companies, corporates, or governments. Deeper understanding of how these organizations can translate the principles of trustworthy AI into practice is needed, including methods for the early detection of errors and a focus on contestability and accountability [54].

Our research provides initial insights into the steps organizations can take to meet the public’s desire for ethical and trustworthy AI systems. Most respondents (57–66%) report they would be more willing to use AI systems if there were mechanisms in place to assure trustworthiness. These mechanisms include independent bodies conducting regular ethical reviews of AI systems, organizational AI ethical codes of conduct, and adhering to AI ethics certification systems and national standards of explainability and transparency. Algorithmic Impact Assessment Tools are being encouraged and used in several jurisdictions [55], and independent audits have been also proposed as a mechanism of AI governance that is actionable and enforceable [56].

As AI systems continue to evolve, so too does the policy landscape. This year, the United States Federal Trade Commission published guidelines for ‘truth, fairness, and equity’ in the use of AI [57], and the European Commission released its recommendations for AI regulation [51]. Both stress the need for transparent AI accountability, with the US guidelines going so far as to say: *“Hold yourself accountable—or be ready for the FTC to do it for you.”* [57]. Ideally forthcoming policy will involve transcontinental cooperation and data protection to avoid fragmentation, preferably moving toward a common global approach. Laws may take many years to take full effect, but organizations should act proactively and preemptively in light of the developing regulatory landscape of AI and citizen expectations.

Given the exponential growth of the AI industry, its global reach, and diverse nature, we argue that quick traction on mitigating the risks of AI systems will require the right policy levers. These levers can incentivize organizations to incorporate trustworthy practices in the development and deployment of AI systems. Without this critical shift, we are unlikely to see the speed or scale required to effectively mitigate the risks and vulnerabilities of AI systems. A rare example of such a policy lever is the EU proposal for an Artificial Intelligence Act (2021), which promotes a scaled, risk-based approach, and sets boundaries on how and when certain high-risk forms of AI may be used. This policy has a requirement that organizations demonstrate implementation of the trustworthy principles for high-risk AI systems to receive the conformity mark to enter the European market [51].

There is precedent for this type of public mandate; organizations are held to account for reporting and managing environmental, social, and governance (ESG) issues related to their operations. We can and should expect this same level of accountability and corporate responsibility with the development and use of AI. Beyond policy levers, achieving trustworthy AI systems requires a whole-of-organization and whole-of-society approach: it cannot be left solely to technical teams [48, 50].
